# Case Report of Recurrent Metastatic Pancreatic Neuroendocrine Tumor with Gastric Invasion: Consequences of Potential Needle-Tract Seeding from Fine-Needle Aspiration

**DOI:** 10.1089/crpc.2016.0012

**Published:** 2016-08-01

**Authors:** Richard Zheng, Sami Tannouri, Harish Lavu

**Affiliations:** ^1^ Department of Surgery, Thomas Jefferson University Hospital, Philadelphia, Pennsylvania.; ^2^ Pancreas, Biliary and Related Cancer Center, Thomas Jefferson University, Philadelphia, Pennsylvania.

**Keywords:** metastatic pancreatic neuroendocrine tumor, PNET

## Abstract

**Background:** Pancreatic neuroendocrine tumors (PNETs) are relatively rare, and data guiding management of metastatic lesions are scarce. Hepatic metastases are most common; here we describe a case of metastatic PNET implanted into the posterior gastric cardia.

**Case Presentation:** This case study describes the progression of a 44-year-old man with a history of pancreatic neuroendocrine tumor (PNET) resected through distal pancreatectomy and splenectomy who developed recurrent disease in his stomach with extension into the left adrenal fossa 17 months after initial resection. He subsequently underwent a total gastrectomy and left adrenalectomy with en bloc resection of this recurrence without complication. Final pathology revealed a morphologically similar PNET with positivity for CAM5.2, chromogranin A, and synaptophysin.

**Conclusion:** The unusual location of his recurrence could suggest that his preoperative endoscopic ultrasound and fine-needle aspiration may have had a role in seeding the posterior gastric wall, highlighting the risk of performing this diagnostic procedure in the setting of suspected pancreatic malignancy.

## Introduction

Pancreatic neuroendocrine tumors (PNETs) are fairly rare, accounting for less than 1–2% of all pancreatic masses. Although they tend to have a more favorable prognosis than pancreatic ductal adenocarcinoma, hepatic metastases are observed in more than 50% of patients with PNETs and are associated with a 5-year survival of only 30–40% when untreated.^1^ Data surrounding treatment of other sites of metastatic spread are limited. Here, we report a case of metastatic PNET in the gastric cardia with extension into the left adrenal fossa, 17 months after distal pancreatectomy and splenectomy. Pre-operative endoscopic ultrasound (EUS) and fine-needle aspiration (FNA) may be implicated in the local implantation of this unusual lesion.

## Case Report

**FIG. 1.**
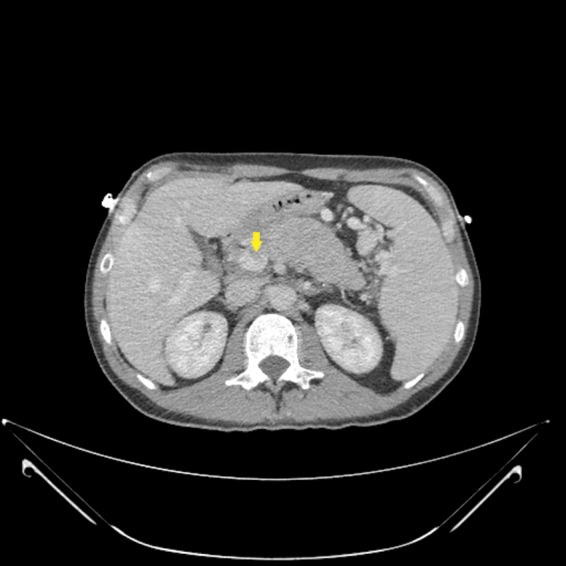
Hypervascular pancreatic body mass with intraluminal enhancement within portal vein consistent with tumor thrombus (arrow).

A 48-year-old man initially presented with lightheadedness, palpitations, and tarry stools. He was found to be anemic with a hemoglobin of 5.5 g/dL. Upper endoscopy revealed nonbleeding gastric varices. Further workup through abdominal CT and MRI demonstrated a large arterial-enhancing pancreatic mass, thought to be a PNET. No metastases were seen. The mass appeared to abut the splenic vein and portosplenic confluence with evidence of thrombus within the lumen of the portal vein (Fig. 1). Endoscopic ultrasound was performed with FNA of the pancreatic mass. Histopathological review of the FNA sample revealed neoplastic cells, positive for Anti-Pan Cytokeratin Antibody, synaptophysin, and CD56, suggesting PNET. An octreotide scan showed focally increased signal at the site of the pancreatic mass, without dissemination.

The patient underwent resection of the tumor through distal pancreatectomy and splenectomy with en bloc portal vein resection and reconstruction as the tumor was directly invading the splenic vein with thrombus extending into the portal vein. The patient tolerated this procedure well and his postoperative course was uncomplicated. Pathology from this initial procedure revealed a grade II PNET with 2/15 adjacent lymph nodes positive for metastatic carcinoma and negative margins with a Ki-67 proliferative index of 15% (grade II).

**FIG. 2.**
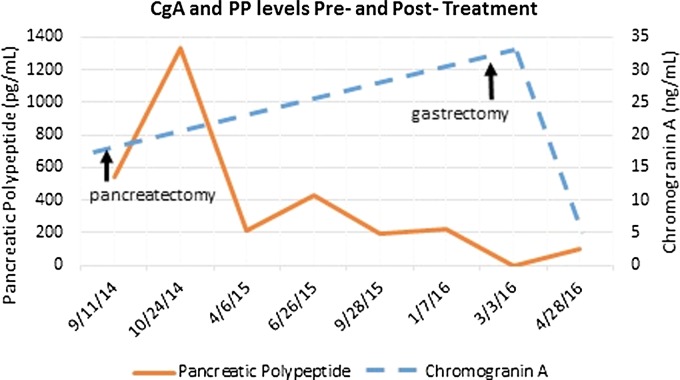
CgA and PP levels before, during, and after treatment course. CgA, chromogranin A; PP, pancreatic polypeptide.

**FIG. 3.**
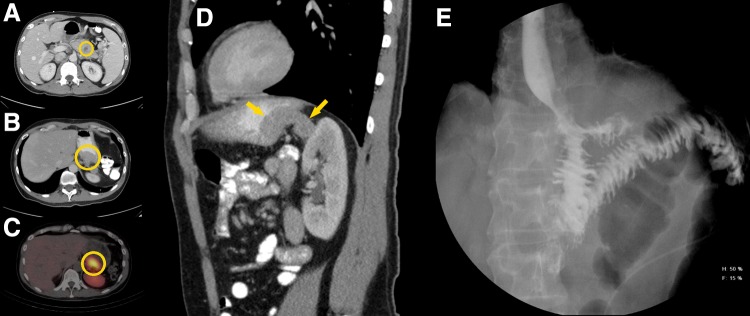
**(A)** Initial abdominal CT scan demonstrating pancreatic body lesion (yellow circle). **(B)** Abdominal CT scan 1 year after distal pancreatectomy and splenectomy with gastric mass visible (yellow circle). **(C)** Octreotide scan with enhancement of the posterior gastric wall (yellow circle). **(D)** Sagittal cut of abdominal CT scan showing gastric mass extending posteriorly (yellow arrows) toward the left adrenal gland. **(E)** Postoperative gastrographin swallow showing anatomical reconstruction through esophagojejunostomy.

Seventeen months after resection, the patient began to have complaints of fatigue, exercise intolerance, and palpitation. He was again found to be anemic with a hemoglobin of 6.6 g/dL. Before the aforementioned initial resection, serum pancreatic polypeptide and chromogranin A levels had been elevated to 547 pg/mL and 15.8 ng/mL, respectively; with the onset of these new symptoms, the pancreatic polypeptide level was found to be only 226 pg/mL, but the chromogranin A level was elevated to 33 ng/mL (Fig. 2). CA 19–9 levels remained normal throughout the patient's full treatment course. Diagnostic endoscopy to localize the source of bleeding revealed a 6 cm malignant-appearing friable mass with central ulceration in the gastric cardia. CT scan findings confirmed the presence of this gastric soft tissue mass and also revealed a posterior nodular extension toward the upper pole of the left kidney (Fig. 3). This lesion demonstrated uptake on octreotide scan, suggesting a PNET origin; a focus of uptake was also noted in the periportal region, suggesting the possibility of a metastatic lymph node. Upon histopathological review of the material retrieved at the endoscopy, the gastric lesion was identified to be morphologically similar to his previous PNET, with positivity for CAM5.2, chromogranin A, and synaptophysin.

**FIG. 4.**
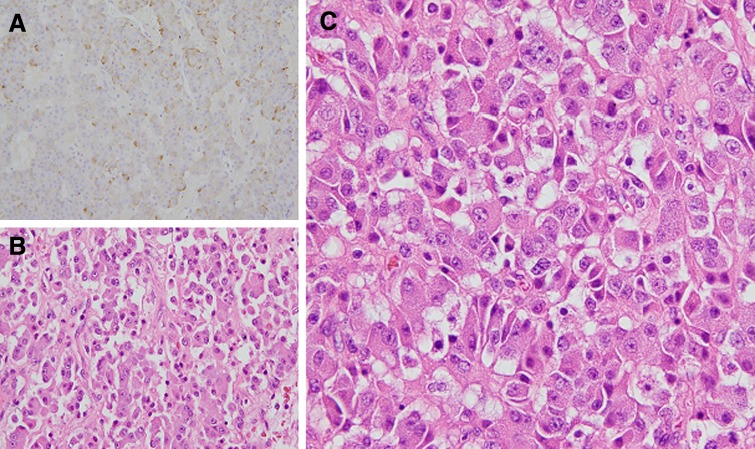
**(A)** Original pancreatic mass with synaptophysin staining, 20× magnification. **(B)** Original pancreatic mass with HE staining, 40× magnification. **(C)** Recurrent gastric mass with HE staining, 40× magnification. HE, hematoxylin and eosin.

After extensive discussion with the patient regarding his treatment options, he opted for reresection through an open total gastrectomy. After exploratory laparotomy and extensive lysis of adhesions, the tumor was noted to invade into the capsule of the left adrenal gland. Both the stomach and the left adrenal gland were removed en bloc. No hepatic lesions were noted. Alimentary tract reconstruction was performed through a Roux-En-Y esophagojejunostomy. The periportal region was skeletonized with removal of all nodal tissue. The patient's postoperative course was uncomplicated. He was ultimately discharged to home on postoperative day 6 on a low-volume clear liquid diet and total parenteral nutrition supplementation. Surgical pathology revealed a pancreatic neuroendocrine carcinoma in the gastric wall and adrenal gland with negative resection margins. The sampled periportal lymph nodes were free of disease. The lesion was again confirmed to be morphologically similar to the patient's previous PNET (Fig. 4). Shortly thereafter, pancreatic polypeptide and chromogranin A normalized to 96 pg/mL and 5 ng/mL, respectively.

## Discussion

PNETs are rare, and metastatic spread linked to FNA is not well documented in the literature. Surgical resection remains the gold standard of treatment for functioning and nonfunctioning tumors. Management of these metastases has not yet been standardized. Surgical management of hepatic metastases through partial hepatectomy has been shown to control symptoms of hormonal hypersecretion in patients with functional tumors and improve overall survival. Hepatic-directed therapy such as transarterial chemoembolization has also been utilized for control of liver metastases.

A case similar to ours was described in 2014. In that report,^2^ a 78-year-old man was found to have a T3N0M0 moderate-to-well differentiated pancreatic adenocarcinoma sampled through EUS/FNA biopsy through the stomach wall. Nine months after resection of the lesion, the patient was found to have a submucosal gastric recurrence upon follow-up endoscopy. This was managed through a subtotal gastrectomy, whereupon final pathology demonstrated a morphologically identical lesion to his initial cancer. In comparison, our lesion had expanded aggressively into the retroperitoneum and appeared to involve the adrenal gland, so a total gastrectomy and en bloc adrenalectomy were required for full resection. Furthermore, as in the previously mentioned case report, FNA played an important role in the preliminary diagnosis of PNET. Given the atypical foci of recurrence in the gastric cardia, this scenario brings to mind the rare phenomenon of needle-tract seeding that has been documented in sarcoma, prostate cancer, hepatocellular carcinoma, and other lesions.^3^

Despite the theoretical fears of needle-tract seeding, however, data validating this phenomenon with regard to some forms of pancreatic cancer are generally limited to case reports.^4,5^ Furthermore, this is the first report of a pancreatic neuroendocrine neoplasm recurrence potentially from needle-tract seeding. A retrospective study of 256 patients who underwent surgical resection of malignant pancreatic neoplasms concluded that undergoing EUS/FNA of these lesions was not associated with a statistically significant increase in the rate of either peritoneal or gastric metastases.^6^ Similarly, use of EUS/FNA for diagnosis of pancreatic cancer was not associated with any difference in survival.^6^ Multiple studies have also corroborated that the use of EUS/FNA for both pancreatic cancer and intraductal papillary mucinous neoplasm sampling was not associated with a statistically significant increase in peritoneal seeding when compared with patients who were not sampled.^7,8^ Alternative approaches for tissue sampling of pancreatic masses have not proven to be of benefit; one study demonstrated an increase in the rate of peritoneal carcinomatosis in patients who had undergone a percutaneous FNA when compared with those who had endoscopic tissue sampling.^9^ Although large-scale data on pancreatic needle biopsy seeding is yet unavailable, it is likely a rare occurrence as needle biopsy of pancreatic lesions is commonplace and gastric metastases are seldom seen. In most cases, the benefits of direct tissue diagnosis outweigh the theoretical risk of needle-tract seeding. However, no matter how remote the chance for seeding may be, it is up to clinicians to be selective in obtaining biopsies of lesions where a tissue diagnosis may change management and not subject a patient to undue risk. In this particular case, one could question the utility of two interventions before the initial operation—the distal pancreatectomy and splenectomy: the FNA done as part of the EUS, and the octreoscan. The patient had a classic appearance of a PNET on CT and MRI, and he was symptomatic from anemia related to splenic vein occlusion. Did the FNA results alter management? Did the octreoscan provide essential information? In this era of cost consciousness in surgical care, we need to be asking these questions.

## Abbreviations Used

EUS: endoscopic ultrasound
FNA: fine-needle aspiration
PNETs: pancreatic neuroendocrine tumors
